# The Role of Anti-calcitonin Gene-related Peptide in Migraine and its Implication in Developing Countries: A Reasonable Option to Consider Despite Higher Cost

**DOI:** 10.7759/cureus.4796

**Published:** 2019-06-01

**Authors:** Ali Akhtar

**Affiliations:** 1 Internal Medicine, Pakistan Air Force Hospital, Islamabad, PAK

**Keywords:** migraine, headache disorder, cgrp antagonist, cgrp monoclonal antibodies

## Abstract

Migraine is among the commonest causes of headache in all ages. It is the second most common cause of neurological disability. Currently, antihypertensive, antidepressants and antiepileptic drugs are reasonable preventable medications for chronic migraine. Despite the higher levels of stigma associated with this disease, fewer attempts were made in past regarding the treatment options for chronic migraine. Recently, a novelty treatment was introduced known as calcitonin gene-related peptide (CGRP) monoclonal antibodies as a possible mechanism for the prevention of migraine attacks. CGRP played an important role in the etiology of migraine headaches and was considered as the major peptide behind the cause of the headache disorders. We have reviewed the benefits of these monoclonal antibodies in terms of their efficacy and adverse effects with the available treatment choices. These drugs showed superior results when compared to the placebo and were considered generally safe in the majority of clinical trials. Earlier versions of CGRP antagonists, known as gepants, were less tolerable due to their tendency to cause liver and cardiovascular complications. Thus, in comparison to the earlier gepants, these CGRP monoclonal antibodies were safer and demonstrated excellent tolerability. Short-term side effects were only limited to mild-moderate injection site rash or pruritus, however, their long-term side effects are still unknown. Despite the higher cost of these drugs, we have analyzed the applicability of this drug in the developing countries. Although the quality-adjusted life year (QALY) gained per cost of the drug is still expensive and majority of people may not afford, its excellent tolerability and less adverse effects should also be considered a reason to implement this drug, particularly for resource-limited countries. Moreover, these medications could also become a prototype for future inventions and creations (cost-effective versions for resource-limited countries). In conclusion, this review suggests that CGRP monoclonal antibodies are safer and excellent alternate option for patients with chronic migraine as it has better efficacy, tolerability, and provides a hope to reduce the stigma associated with migraine. All these benefits should be the deciding factors when opting for this treatment and the decision should not be made solely on the socioeconomic status.

## Introduction and background

Migraine is one of the most common neurological disorders which happens to affect almost all age groups. The most common presenting symptoms are severe headaches which are often accompanied or triggered by strong visual light, sound or odour. The discovery of calcitonin gene-related peptide (CGRP), roughly 34 years ago, was thought to be one of the major peptides that had specific role in the regulation of cerebral blood vessels [1]. In 1992, an attempt was made to understand the role of sumatriptan and dihydroergotamine on the suppression of CGRP on the animal models and humans [2]. To further identify the role of CGRP as a culprit behind the migraine headache in the migraineurs, a double blind cross-over study was conducted in 2002 which reported severe headaches in patients following the administration of human αCGRP [3]. Hence, the role of CGRP was defined as the cause behind episodic migraine headache.

CGRP and migraine pathophysiology

CGRP, a 37-amino acid peptide, is the predominant peptide responsible for cerebral and peripheral blood vessel dilatation [4]. It exists in α and β isoforms. In the brain, the αCGRP is mainly expressed in the regions of trigeminovascular system, whereas β isomer is mainly found in the enteric nervous system [5]. However, while cerebral vessel dilation is one of its major effects, nociception, uptake of glucose and the stimulation of glycolysis in skeletal muscles are considered its other effects [6, 7].

The role of CGRP in migraine pathophysiology had been the motivation behind the innovation of the drug called CGRP antagonists. The first designer drug, olcegepant (BIBN4096BS), had high affinity and selective CGRP antagonist [8]. These CGRP antagonists resulted in liver toxicity for which their use was proposed to be discontinued. However, due to their adverse-effects, new and modified drugs called CGRP receptor antibodies were created recently. These newly developed drugs worked well outside central nervous system (CNS) (in particular, meninges) and they did not cross blood-brain barrier (BBB), whereas gepants and triptans crossed BBB but only to a minor degree [9, 10]. This review will highlight efficacy of CGRP receptor blockers in terms of its benefits and associated adverse effects. Nevertheless, our main focus of this review will be its consideration for developing countries despite its higher cost.

## Review

Efficacy of gepants: based on two hours pain-free interval studies

Gepants generally provide relief to the patients with headache associated with migraine. In 2016, out of all gepants, telcagepant was studied in the majority of clinical trials as the prototype drug and showed significantly better results when compared to placebo or alternate choices [11]. However, when compared with the variety of drugs belonging to the triptans family, telcagepant, BI44370, and rimegepant showed no significant difference in terms of their efficacy [12-14]. Interestingly, two newer drugs, ubrogepant and rimegepant, showed lower efficacy than triptans and simple analgesics. Their low efficacy was postulated due to their slower oral absorption, and in decreased therapeutic gain when compared to telcagepant [15].

Efficacy of antibodies against CGRP: based on phase III and phase II clinical trials

These antibodies against CGRP or its receptor have played pivotal role in the management of migraine. Their action out of CNS (as they do not cross BBB) and in particular, in the trigeminal system, is responsible for the relief of migraine attacks [10]. Currently, there are four monoclonal antibodies, i.e., eptinezumab, fremanezumab, galcanezumab and erenumab that are being studied for chronic and episodic migraine [16]. Out of these, galcanezumab and fremanezumab published phase III trials, whereas erenumab despite better results was only tested in phase II trials. Galcanezumab showed slight better results when compared to fremanezumab in the chronic migraine (defined as headache for equal or more than 15 days). Nevertheless, out of all these drugs, best results were seen with trial of eptinezumab which showed a reduction in monthly number of headaches (-8 days) in chronic migraine [16]. Table 1 provides an overview of the efficacy of current monoclonal antibodies (mAbs) in management of chronic migraine in terms of mean reduction of headache days per month.

**Table 1:** The efficacy of current monoclonal antibodies in management of chronic migraine in terms of mean reduction of headache days per month. CGRP: Calcitonin gene-related peptide

CGRP Monoclonal Antibodies	Mean Reduction of Headache Days per Month
Eptinezumab	-8
Fremanezumab	-4.6
Galcanezumab	-4.8
Erenumab	-6.6

Headache disorder in developing countries: migraine being the most documented literature over the past decade

A study was published in 2008 that gathered data from major developing countries from 1997 to 2006 using the term “headache” from low, lower middle, upper middle and higher socioeconomic countries (classification based on World Bank categorization). Out of all the known causes of headaches, migraine was the focus of majority publications representing 48.9% of the data, whereas non-migraine pain constituted about 26.4%. Only one-third (29.5%) of the data showed articles for the treatment of headache [17]. The World Health Organization (WHO) ranks migraine as the third most common headache in the world and second most disabling neurological condition [18]. Considering the predominant cause of headaches in developing countries is migraine yet only one-third of the data discusses its treatment. The WHO has identified three barriers to effective care for headaches, and in particular, migraine [18]. Table 2 summarizes three barriers to effective care.

**Table 2:** Three barriers to the effective treatment of headache disorders by WHO.

World Health Organisation barriers to effective migraine treatment		
Lack of knowledge among healthcare providers: Healthcare providers are not well trained to diagnose and treat headache disorders.	Poor awareness: Headaches are not perceived as serious threat due to its episodic nature.	Direct and indirect costs due to delay seeking treatment: Despite advancement, people often delay treatment due to costly investigations pertaining to accurate diagnosis.

The role of CGRP monoclonal antibodies in developed countries: why is it still a reasonable choice despite being an expensive treatment?

According to the Institute for Clinical and Economic Review (ICER) report, CGRP monoclonal antibodies are costly, but they provide more migraine-free days, and increased QALYs when compared to no treatment in both episodic and chronic migraine. The estimated cost of each migraine-free day with erenumab or fremanezumab is between $130 and $340, in comparison with no treatment [19]. Considering the high cost of these medications, these drugs were thought to divide the lower socioeconomic societies because of their higher market value, and specifically when majority of the developing countries are still under the circumstances of choosing between their basic necessities. As mentioned by Burch and Rayhill, currently there are only about 20% of patients who could gain benefits of erenumab despite considering its skyrocketed price without becoming a burden on the society. For a population to become eligible for CGRP monoclonal antibodies at cost-effective price range, 75% reduction in the price, estimated $2200 per year, would be required [20]. Nevertheless, despite enormous prices of these drugs we have summarised three reasons for this novel drug that might benefit people in the poverty with migraine at the same prices.

Firstly, CGRP monoclonal antibodies have shown to achieve positive impact on the QALY or DALY (disability-adjusted life year), which is valuable for someone who has the disease [19]. The term QALY is used to measure the burden of certain disease considering its efficacy in treatment and overall cost (1 QALY is equal to one year of healthy life). Despite outrageous costs of erenumab and fremanezumab, their costs per QALY gained were reported $90,000 and $120,000 in cases of chronic migraine, which are fairly reasonable considering its vital impact in the addition of an extra healthy life year [19]. Based on the figures published by ICER, the importance of QALY cannot be ignored due to the expensive treatment. Secondly, besides higher cost of these medications, they exhibit excellent tolerability. In comparison with the gepants, these drugs are devoid of major side effects such as hepatotoxicity or cardiovascular complications (owing to their vasodilatory properties), with the exception of mild-moderate injection site reaction [6]. CGRP monoclonal antibodies is also considered a reasonable alternate treatment for patients in which earlier treatment has failed [19]. Lastly, migraine is considered a stigmatized disease and unfortunately, anti-stigma efforts were not made at the same pace as were made in the other disorders such as epilepsy. Therefore, proper allocation of resources and introduction of new interventions could be one way to overcome the stigma associated with chronic migraine even in the developing countries. More ways to reduce stigma can be sort through spreading awareness, education, and better policy making strategies [20, 21].

Other aspects of CGRP antagonists

There are a slight number of adverse effects that could arise as a result of CGRP monoclonal antibodies. Firstly, there is a risk of development of a mild-moderate injection site reaction. More common adverse effects for CGRP monoclonal antibodies also included pruritus and erythema [22]. However, no severe adverse effects were reported during clinical trials [6]. Their scarce oral bioavailability led to only parenteral route of administration, which also means longer half-life of the drug [23]. One disadvantage to this route is the need of healthcare provider for the intravenous administration. Secondly, there are no documented long-term side effects of CGRP monoclonal antibodies and its risks are still unknown. However, as stated by the American Headache Society Annual meeting in 2019, in several years more results of clinical trials will be published which will demonstrate long-term safety and its physiological effects.

Current treatment for migraine when compared to CGRP (receptor) blockers

Current preventative treatment options for migraine include antihypertensive drugs, antidepressants and antiepileptic medication. In contrast to CGRP (receptor) antagonists, these have all been developed for other diseases rather than migraine and a reduction in mean monthly frequency of 50% or more is estimated. However, other measures are also emphasized but not limited to keeping headache diaries, education, and adherence to prophylactic treatment [24].

## Conclusions

We have reviewed the benefits and drawbacks of CGRP receptor blockers, although their long-term side effects are still unknown. Double-blind placebo studies have demonstrated favorability and superior results in terms of their efficacy, minimal adverse effects and a suitable alternative choice among chronic migraineurs, even in the resource-limited countries where only about a quarter of literature has been published on the treatments of migraine headaches. Although the higher cost of these medications is still considered a major hindrance particularly in the developing countries, the cost per QALY gained at the current prices alongside with its minimal side effects such as a mild injection site reaction is also reasonable. One must realise the fact that due to minimal adverse effects of these drugs, the cost spent on dealing with long-term side effects (liver damage and cardiovascular complications) associated with the conventional treatment such as gepants, is more than the cost spent on these newer treatments. Moreover, out of all the CGRP receptor mAbs currently in the market, we reviewed that the best results were seen with eptinezumab, which showed a reduction of eight days in monthly number of headaches in chronic migraineurs. Currently, future studies are being planned to identify long-term side effects of these drugs, which may then further clarify their risks and benefits as opposed to the current treatment. Speaking in favour of CGRP receptor blockers, these innovative drugs are currently available in high-end markets. The overly stigma of migraine has finally led to the developments of these drugs which will reduce the level of migraine stigma in future. This break-through innovation in the treatment of chronic migraine will surely set a threshold for more advance, and cheaper versions for resource-limited countries.
